# MDMA treatment paired with a trauma-cue promotes adaptive stress responses in a translational model of PTSD in rats

**DOI:** 10.1038/s41398-022-01952-8

**Published:** 2022-05-03

**Authors:** Shira Arluk, Michael A. Matar, Lior Carmi, Oded Arbel, Joseph Zohar, Doron Todder, Hagit Cohen

**Affiliations:** 1 Department of Psychology, Ben-Gurion University of the Negev, Beer-Sheva, Israel; 2 Beer-Sheva Mental Health Center, Ministry of Health, Anxiety and Stress Research Unit, Faculty of Health Sciences, Ben-Gurion University of the Negev, Beer Sheva, Israel; 3 Post-Trauma Center, Sheba Medical Center, Tel Aviv, Israel; 4 Beer-Sheva Mental Health Center, The Mindfulness Clinic, Beer Sheva, Israel

**Keywords:** Depression, Physiology

## Abstract

MDMA (3,4-methylenedioxymethamphetamine), a synthetic ring-substituted amphetamine, combined with psychotherapy has demonstrated efficacy for the treatment of chronic posttraumatic stress disorder (PTSD) patients. This controlled prospective study aimed to assess the bio-behavioral underpinnings of MDMA in a translational model of PTSD. Rats exposed to predator-scent stress (PSS) were subjected to a trauma-cue at day 7 shortly after single-dose MDMA injection (5 mg/kg). The elevated plus maze and acoustic startle response tests were assessed on day 14 and served for classification into behavioral response groups. Freezing response to a further trauma-reminder was assessed on Day 15. The morphological characteristics of the dentate gyrus (DG) and basolateral amygdala (BLA) were subsequently examined. Hypothalamic–pituitary–adrenal axis and 5-hydroxytryptamine involvement were evaluated using: (1) corticosterone measurements at 2 h and 4 h after MDMA treatment, (2) Lewis strain rats with blunted HPA-response and (3) pharmacological receptor-blockade. MDMA treatment was effective in attenuating stress behavioral responses only when paired with memory reactivation by a trauma-cue. The effects of the treatment on behavior were associated with a commensurate normalization of the dendritic cytoarchitecture of DG and BLA neurons. Pretreatment with RU486, Ketanserin, or Pindolol prevented the above improvement in anxiety-like behavioral responses. MDMA treatment paired with memory reactivation reduced the prevalence rate of PTSD-phenotype 14 days later and normalized the cytoarchitecture changes induced by PSS (in dendritic complexities) compared to saline control. MDMA treatment paired with a trauma-cue may modify or update the original traumatic memory trace through reconsolidation processes. These anxiolytic-like effects seem to involve the HPA axis and 5-HT systems.

## Introduction

Recent clinical studies with 3,4-methylenedioxymethamphetamine (MDMA) in people diagnosed with treatment-resistant posttraumatic stress disorders (PTSD) reported durable remission of PTSD symptoms and prevalence [1–10]. Further, meta-analyses found that MDMA-assisted psychotherapy is a safe, effective, and durable treatment for individuals with PTSD [11–14]. Moreover, MDMA achieved breakthrough therapy designation status from the Food and Drug Administration for treatment-resistant PTSD patients in 2017 [15].

MDMA is a synthetic analog of amphetamine and mescaline stimulants and hallucinogens. It emerged as a popular recreational drug because it can induce strong feelings of euphoria, empathy, and connection to others [16–18]. Its neuropharmacology is complex, with potent effects on serotonin (5-hydroxytryptamine, 5-HT) [18–20], dopamine [21], and norepinephrine [17], as well as the release of cortisol (corticosterone in rodents), oxytocin, prolactin and arginine vasopressin [21–23]. However, the underlying mechanisms by which MDMA-based psychotherapy reduces PTSD symptoms are not understood.

The overall aim of this study was to assess the effects of MDMA on behavioral responses to predator-scent stress (PSS) in a controlled, prospective animal model of PTSD. In this model, populations of exposed rodents are classified according to the degree of their individual behavioral response using standardized ‘cut-off behavioral criteria’ (CBC), creating three distinct groups: ‘extreme behavioral response’ (EBR) and ‘minimal behavioral response’ (MBR) at the extremes, and a middle group of ‘partial behavioral responders’ (PBR) [24–29]. The relative prevalence rates of individuals displaying the different degrees of disrupted behavior provide an indication of the potential efficacy of the ‘treatment’ under study.

The goal of this study was threefold: (1) to provide an evaluation of the effect of MDMA in an animal model of PTSD. To this end, we investigated whether treatment with MDMA, when administered adjunctively to trauma-cue (memory reactivation) or alone, would (1.1) reduce predator-scent stress (PSS)-induced anxiety-like responses and hyperarousal; (1.2) shift the prevalence rates of extreme responders (PTSD-phenotype) towards partial and/or minimal responders; and (1.3) affect the PSS-induced morphological damage in the hippocampus and basolateral amygdala (BLA) and (2) to assess whether glucocorticoid receptors (GR) are involved in the therapeutic effects of MDMA treatment paired with a trauma-cue. We examined the effects of MDMA paired with a trauma-cue on behavioral stress responses in Lewis rats, characterized by an hypoactive and hyporeactive HPA response and greater susceptibility to experimentally induced PTSD-phenotype [30] or using RU486 pre-treatment as pharmacological tools to outbred Sprague-Dawley rats. (3) to assess whether 5-HT_1A,_ 5-HT_2A_ and 5-HT_2C_ receptors are involved in the therapeutic effects of MDMA treatment. To this end, a controlled, prospective trial examined the effects of 5HT_2_ and 5-HT_1A_ and 5-HT_2C_ receptor antagonists, Ketanserin, Pindolol and SB242084, respectively, on behavioral stress responses.

## Materials and methods

All treatment and testing procedures were approved by the Animal Care Committee of Ben-Gurion University of the Negev, Israel.

### Animals

For all experiments, 196 Adult male Sprague-Dawley rats and 26 adult male Lewis (Lew/Crl/CrlBR) rats weighing 200–250 g were used. Rats were habituated to housing conditions for 10 days, housed two/cage in a vivarium with stable temperature and a reversed 12-h light/dark cycle (lights off: 19:00), with unlimited access to food and water. All testing was performed during the dark phase in dim red-light conditions. All experiments and measurements handled by an experimenter blinded to groups conditions.

### Experimental design

We conducted four experiments to assess the effects of MDMA in an established rat model. In experiment 1 (*N* = 80), rats were exposed to a PSS or sham-PSS. Seven days thereafter, MDMA (5 mg/kg) or saline were administered 30 min before exposure (to ensure a peak level during the 1–6 h time window of memory vulnerability) to a trauma-cue (see below) and locomotor activity (LMA) was assessed for 10 min [Assessments of motor activity serve as efficient procedures for gauging in vivo activity of psychostimulants, including MDMA [31]]. Seven days thereafter rats were behaviorally assessed in the elevated plus maze (EPM) and acoustic startle response (ASR) tests at Day 14. These data subsequently served for retrospective classification into behavioral response groups. The prevalence rates of extreme, partial, and minimal behavioral responses were assessed. One day later (day 15), rats were again exposed to a situational reminder (fresh, unused cat litter) and freezing behavior reassessed [To model the disproportionate psychophysiological response to trauma reminders, a stimulus that is not intrinsically threatening but is, however, a clear-cut reminder of the traumatic stressor]. On Day 16, rats were sacrificed, and their brains collected for morphological staining. To investigate the hormones associated with MDMA, 39 rats were sacrificed 2 h (*n* = 20) and 4 h (*n* = 19) after injection, and their blood collected for measurement of corticosterone concentrations. In experiment 2 (*N* = 54), which aimed to elucidate the potential anxiolytic-like effect of MDMA on the underlying reconsolidation process, rats were exposed to PSS and seven days thereafter, MDMA or saline were administered with or without a trauma-cue. Another 2 groups of rats were exposed to PSS and six days thereafter, MDMA or saline were administered, one day before exposure to the situational reminder (i.e., MDMA treatment was unpaired from the reminder). Behavioral parameters were assessed as described in experiment 1. In experiment 3 (*N* = 26), which aimed to explore the role of glucocorticoids in MDMA-induced anxiolytic-like behavioral stress responses, we used the same protocol as in experiment 1 but used Lewis rats which are characterized by an hypoactive and hyporeactive HPA response and display greater susceptibility to experimentally induced PTSD-phenotype [30]. Experiment 4 (*N* = 36) was designed to evaluate the behavioral effects of pharmacological manipulation of GR, 5-HT_2A_, 5-HT_1A_ and HT_2C_ levels prior to MDMA (5 mg/kg) injection. To this end, rats were exposed to PSS. Seven days later, GR antagonist mifepristone (Ru486) (7.5 mg), 5-HT_2A_ R antagonist (RA) Ketanserin (5 mg/kg), 5-HT_1A_.

RA (Pindolol) (0.3 mg/kg), 5-HT_2C_ RA SB242084 (3 mg/kg) or saline were administered 60 min before MDMA injection, which was administered 30 min before a brief trauma-cue session. Behavioral parameters were assessed as in experiment 1. The experimental design used for each of these experiments is schematically depicted in the respective figures.

### Predator-scent stress

Rats were individually placed on well-soiled cat litter, used for 2 days by a cat and sifted for stools [24–29]. Rats were exposed to the litter for 10 min in a plastic cage (inescapable exposure) placed on a yard’s paving stone in a closed environment. Sham-PSS was administered under similar conditions, but rats were exposed to fresh, unused cat litter.

### Drugs

MDMA (5 mg/kg), Ketanserin (5 mg/kg), SB242084 (3 mg/kg), Pindolol (0.3 mg/kg) and RU486 (7.5 mg, approximately 30 mg/kg) were purchased from Sigma-Aldrich Co. (Sigma-Aldrich, Israel). All drugs were dissolved in saline (0.9% NaCl) and injected intraperitoneally (i.p.) in a 250 µl volume. Based on the literature [32–34] and in considering our preliminary study which didn’t find any significantly differences between saline and MDMA 3 mg/kg in LMA and because of the possibility of neurotoxicity produced by higher dose of 10 mg/kg [35], the 5 mg/kg dose was chosen for this study. The doses of ketanserin and SB242084 were selected based on Shinoda’s report [36]. The dose of Pindolol was selected based on Nash’s report [22]. The dose of RU486 was selected based on our previous study [37]. All pre-treatments were administered i.p. 60 min prior to MDMA injection.

### Contextual freezing measurement

The situational reminder consisted of placing the animals on fresh, unused cat litter for 10 min to mimic the context of the initial exposure experience. Freezing behavior during situational reminder was defined as the absence of all movement except for respiration [38]. Behavior was recorded using an overhead video camera and scored for immobility (freezing) and the total distance moved.

### Behavioral measurements

Behaviors of rats were assessed using EPM and ASR, as described previously [24–29]. Detailed protocols are described in Supplementary Information 1.1.

### Cut-off behavioral criteria model

Individual rats were classified according to the degrees to which individual behavior was affected by a stressor. The classification of individual rats was based on the premise that extremely compromised behavior in response to the priming trigger is not conducive to survival, inadequate and maladaptive, and thus represents a pathological degree of responses [24–29]. Please see Supplementary Information 1.2 for a detailed explanation of the criteria.

### Corticosterone sampling

Corticosterone (in plasma or urine) was measured with a DSL-1081000 ELISA kit according to manufacturer instructions (Diagnostic Systems Laboratories, Webster, TX) by a person blind to experimental procedures. The sensitivity of the corticosterone assay is 12.5 μg/L. Within-assay variation is <10% and between-assay variation <14% at 100 μg/L. All samples were measured in duplicate.

### Golgi–Cox staining

Twenty-four hours after the behavioral tests animals were deeply anesthetized and perfused intracardially with 0.9% saline. The brains were immediately dissected and processed as described below. Tissue was prepared by using the rapid Golgi kit (FD Neurotechnologies, USA) according to manufacturer’s instructions. Please see Supplementary Information 1.3 for a detailed explanation of the criteria.

### Statistical analyses

For behavioral tests, statistical analyses were performed with a two-way analysis of variance (ANOVA) with PSS and Treatment as independent factors. For the corticosterone concentration and Sholl analysis, a two-way repeated measured (RM)ANOVA was used. Post-hoc Bonferroni tests were used to examine differences between individual groups. In addition, behavioral data were transformed to percentages using the cut-off behavioral criteria model: the prevalence of affected rats as a function of the rat group was tested by using cross-tabulation and nonparametric Chi-squared tests. All nonparametric analyses were performed on raw data (and not on percentage).

## Results

**Fig. 1:**
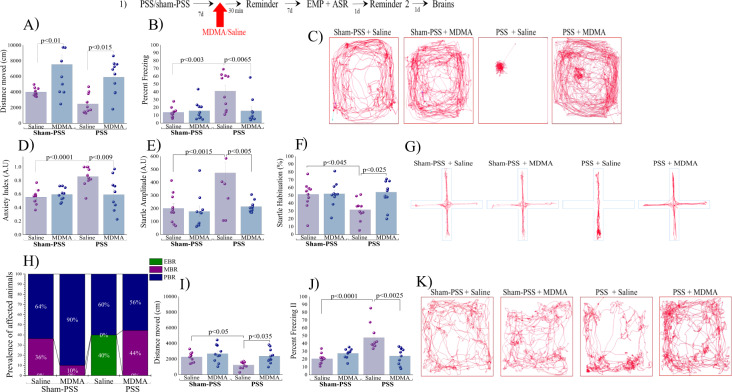
MDMA treatment 30 min before trauma-cue at 7 days after trauma, attenuates behavioral stress responses The top panel (1) depicts the experimental protocol. Vertical arrows represent intraperitoneal MDMA (5 mg/kg) or saline injection. Rats were exposed for 10 min to predator-scent stress (PSS) or to sham-PSS on day 0. On day 7, rats received MDMA (sham-PSS + MDMA: *n* = 10; PSS-exposed + MDMA: *n* = 10) or saline (sham-PSS + saline: *n* = 11; PSS-exposed + saline: *n* = 10) and 30 min later were exposed to a trauma-cue for 10 min. During this time **A** LMA (distance moved; measured in cm); **B** Percent freezing response; **C** Representative patterns of locomotor activity (cumulated values) in all groups. Behavioral measurements (EPM and ASR) were performed on day 14 and freezing behavior on day 15. **D** Anxiety index, which integrates the measured EPM behavioral measures; **E** Startle amplitude in the ASR paradigm: **F** Percentage of startle habituation in the ASR paradigm. **G** Representative accumulated movement track during a trial. **H** Prevalence of extreme behavioral response (EBR) vs. partial behavioral response (PBR) and minimal behavioral response (MBR) rats (in percentages). Significant differences were found between groups in the prevalence rates of individuals displaying an extreme, minimal, and partial behavioral response (Pearson *χ*^2^ = 18.85, df = 10, *p* < 0.0045). Finally, effect of trauma-cue at day 15 on LMA **I** (distance moved; measured in cm); and **J** Percent freezing response. **K** Representative patterns of locomotor activity (cumulated values) in all groups. The experiments described below were performed with two different cohorts of animals; 20 rats were run in one experimental design (5 rats/each group), and 21 rats in another experimental design (5–6 rats/each group). Bars represent group means ± SEM and percentages.

Experiment 1: MDMA treatment attenuates behavioral stress responses: First, we measured the LMA during exposure to the trauma-cue, in rats treated with MDMA vs. saline to gauge the in vivo psychostimulant effect of MDMA. We observed that MDMA treatment increased the total distance moved in the arena (in both sham-PSS and PSS-exposure groups) as compared to controls (*p* < 0.01 and *p* < 0.015, respectively; Fig. 1A–C) [Two-way ANOVA: *PSS-exposure effect:* *F*(1,36) = 4.6, *p* < 0.04; *Treatment effect:* *F*(1,36) = 22.6, *p* < 0.0001; *Exposure–treatment interaction:* NS]. Repetitive or stereotypical movements, such as head weaving, sniffing, proptosis, and forepaw treading, were not observed during the tests. During trauma-cue experiment, MDMA treatment reduced the freezing behavior (immobility) induced by prior PSS as compared to exposed rats treated with saline (*p* < 0.0065; Fig. 1B; [Two-way ANOVA: *PSS-exposure effect:* *F*(1,36) = 7.2, *p* < 0.015; *Treatment effect*: *F*(1,36) = 5.4, *p* < 0.03; *Exposure-treatment interaction:* *F*(1,36) = 7.4, *p* < 0.01]). Taken together, MDMA treatment produced a significant hyper-locomotor response under all conditions (stimulant effect).

Using our validated animal model of PTSD [24, 27–29], we next assessed the long-term behavioral effects of a single dose of either MDMA or saline injected 30 min before exposure to a trauma-cue. Seven days later (Day 14), we assessed the behavior of these rats in two well-established, stress-related paradigms—the EPM and the ASR.

### Elevated plus maze

MDMA treatment reduced the anxiety index induced by prior PSSexposure as compared to PSS-exposed control rats treated with saline (*p* < 0.009); Fig. 1D–G; [Two-way ANOVA: *PSS-exposure effect:* *F*(1,36) = 8.9, *p* < 0.0055; *Treatment effect:* *F*(1,36) = 5.0, *p* < 0.035; *Exposure-treatment interaction effect:* (*F*(1,36) = 9.3, *p* < 0.0045]).

Please see Supplementary Information 2.1 for all EPM parameters.

### Acoustic startle response

MDMA treatment reduced the startle amplitude and increased percentage of startle habituation induced by prior PSS-exposure as compared to PSS exposed control rats treated with saline (*p* < 0.005 and *p* < 0.025, respectively; Fig. 1E, F; [Two-way ANOVA: Startle amplitude: *PSS-exposure effect:* *F*(1,36) = 10.16, *p* < 0.003; *Treatment effect:* *F*(1,36) = 8.5, *p* < 0.0065; *Exposure-treatment interaction effect*: *F*(1,36) = 5.8, *p* < 0.025; Startle habituation: *Treatment effect:* *F*(1,36) = 5.5, *p* < 0.025; *PSStreatment interaction effe*ct: *F*(1,36) = 4.8, *p* < 0.04]). These findings indicate that a single MDMA injection paired with a trauma-cue significantly normalized the startle amplitude and habituation induced by PSS.

### Relative prevalence rates of behavioral responses according to cut-off behavioral criteria model

We used the results of these paradigms in a statistically validated cut-off behavioral criteria model [27–29] (Supplementary Information 1.2) to functionally classify rats according to their overall stress-related behavior. There were significant differences in the prevalence rates of individuals displaying EBR among groups (Pearson *χ*^2^ = 13.33, df = 3, *p* < 0.004; Fig. 1H). The prevalence of EBR among PSS-exposed rats injected with saline was 40.0% of the total population and differed significantly from the sham-exposed group (*χ*^2^ = 5.44, *p* < 0.02), and from exposed rats treated with MDMA (*χ*^2^ = 4.56, *p* < 0.035), among which there were no EBR cases. There were no MBR cases among PSS-exposed rats injected with saline, a result that significantly differed from sham-exposed rats (*χ*^2^ = 4.49, *p* < 0.035). The prevalence of MBR among PSS-exposed rats treated with MDMA was 44.0% of the total population and differed significantly from exposed rats treated with saline (*χ*^2^ = 5.63, *p* < 0.02), where there were no MBR cases. There were no significant differences in PBR prevalence among groups. These analyses indicate that MDMA paired with a trauma-cue elicits a significant shift towards less extreme behavioral disruption in rats.

### Freezing behavior at day 15

MDMA treatment increased the total distance moved (Fig. 1I–K) and decreased immobility (Fig. 1J, K) induced by prior PSS-exposure as compared to PSS-exposed rats treated with saline (*p* < 0.035 and *p* < 0.0025, respectively; Fig. 1I–K; [Two-way ANOVA: Distance moved: *PSS-exposure effect:* *F*(1,35) = 15.2, *p* < 0.00045; *Exposure-treatment interaction effects:* *F*(1,35) = 14.5, *p* < 0.00055; Percent freezing: Two-way ANOVA: *PSS-exposure effect:* *F*(1,35) = 6.2, *p* < 0.02; Treatment effect: *F*(1,35) = 8.5, *p* < 0.0065; *Exposure-treatment interaction effects: = NS*]). At day 15, in the sham-PSS group, no differences were observed in percent freezing and distance moved between saline- and MDMA-treated rats, indicating that MDMA had no long-term intrinsic effects on LMA, distance moved or freezing behavior. These findings indicate that a single MDMA injection paired with a trauma-cue significantly attenuates the memories of threat and intense fear 7 days after injection.

Morphology at Day 16: To gain insights into the cytoarchitecture changes by which MDMA attenuated the PTSD-related responses, brains were harvested 24 h later (Day 16) and neurons from DG subregion and BLA were reconstructed and subjected to Sholl analysis.

#### DG granular neurons

**Fig. 2:**
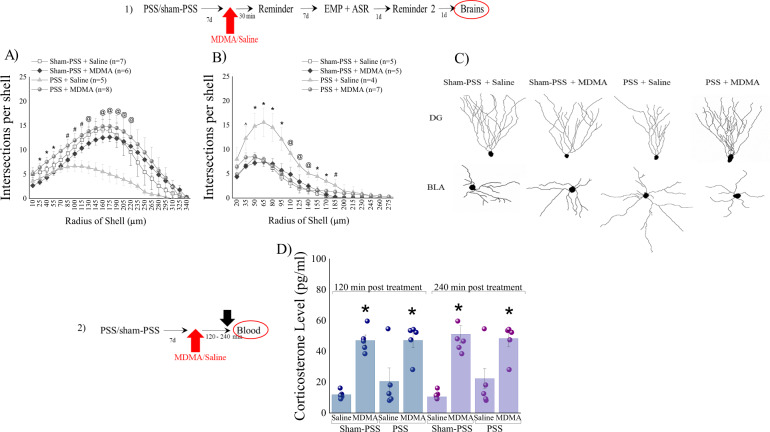
MDMA treatment paired with a trauma-cue at 7 days after trauma, normalized morphological indicators. The top panel (1) depicts the experimental protocol. Vertical arrows represent intraperitoneal MDMA (5 mg/kg) or saline injection. Rats were exposed for 10 min to predator-scent stress (PSS) or to sham-PSS on day 0. On day 7, rats received MDMA (sham-PSS + MDMA: *n* = 10; PSS-exposed + MDMA: *n* = 10) or saline (sham-PSS + saline: *n* = 11; PSS-exposed + saline: *n* = 10) and 30 min later were exposed to a trauma-cue for 10 min. On Day 16, rats were sacrificed and their brains collected for morphological staining. **A** Sholl analysis for intersections per 15-μm radial unit distance of dentate gyrus granule cells from the suprapyramidal blade. * Sham-PSS + MDMA ≠ PSS + MDMA, *p* < 0.05. ^#^PSS + saline ≠ PSS + MDMA, *p* < 0.05. ^@^PSS + saline ≠ PSS + MDMA, sham-PSS + saline, *p* < 0.05. **B** Sholl analysis for intersections per 15-μm radial unit distance of pyramidal neurons of the basolateral amygdala. ^^^PSS + saline ≠ sham-PSS + saline & sham-PSS + MDMA, *p* < 0.03. * PSS + saline ≠ sham-PSS + saline & sham-PSS + MDMA & PSS + MDMA, *p* < 0.03. ^@^PSS + saline ≠ sham-PSS + saline & PSS + MDMA, *p* < 0.05. ^#^PSS + saline ≠ sham-PSS + saline, *p* < 0.04. **C** Computer-generated reconstructions of dendritic trees from granule cells and pyramidal cells in all groups. (2) depicts the experimental protocol. Vertical arrows represent intraperitoneal MDMA (5 mg/kg) or saline injection. Corticosterone concentrations (pg/ml) measured at 120- and 240 min post MDMA treatment in sham-PSS treated with saline (Sham-PSS + saline, *n* = 10) or MDMA (Sham-PSS + MDMA, *n* = 10), PSS-exposed animals treated with saline (PSS + saline, *n* = 10), or PSS-exposed treated with MDMA (PSS-MDMA, *n* = 9). **D** corticosterone concentrations** (pg/ml) were measured. The experiments described below were performed with two different cohorts of animals; 20 rats were run in one experimental design, and 19 rats in another experimental design. Bars represent group means ± SEM.

MDMA treatment elevated the number of dendritic intersections with each sphere at Sholl radii between 25–220 μm induced by prior PSS-exposure as compared to PSS-exposed rats treated with saline (*p* < 0.05; Fig. 2A; [Threeway RM-ANOVA: *PSS-exposure effect:* *F*(1,505) = 8.8, *p* < 0.0035; *Treatment effect*: *F*(1,505) = 57.4, *p* < 0.0001; *Radius effect:* *F*(22,505) = 19.4, *p* < 0.0001; *Exposure-Treatment interaction effects:* *F*(1,505)=68.2, p < 0.00001]). No differences between sham-exposed animals treated with MDMA or saline or the PSS-exposed group treated with MDMA were detected at any radii. Please see Supplementary Information 2.2 for morphological parameters.

#### Pyramidal neurons of the BLA

MDMA treatment reduced the number of dendritic intersections with each sphere at Sholl radii between 35–220 μm induced by prior PSSexposure as compared to PSS-exposed rats treated with saline (*p* < 0.05; Fig. 2B; [Threeway RM-ANOVA: *PSS-exposure effect:* *F*(1,340) = 90.8, *p* < 0.0001; *Treatment effect:* *F*(1,340) = 40.2, *p* < 0.0001; *Radius effect*: *F*(19,340) = 70.6, *p* < 0.0001; *Exposure-Treatment interaction effects:* *F*(1,340) = 60.3, *p* < 0.00001; *Exposure-Radius interaction effects*: *F*(19,340) = 2.6, *p* < 0.0003; *Treatment-Radius interaction effects*: *F*(19,340) = 2.7, *p* < 0.00025; *Exposure-Treatment-Radius interaction effects*: *F*(19,340) = 3.6, *p* < 0.00001]). No differences between sham-exposed animals treated with MDMA or saline or the PSS-exposed group treated with MDMA were detected at any radii.

Please see Supplementary Information 2.2 for morphological parameters.

### Corticosterone

To investigate the hormones associated with MDMA, we examined whether -administering MDMA 30 min before exposure to a trauma-cue, 7 days after exposure to PSS modifies the activation of the hypothalamic–pituitary-adrenal (HPA) axis, as reflected in circulating corticosterone levels. At both time points (2 and 4 h), rats treated with MDMA (sham-PSS and PSS exposure) displayed significantly higher serum corticosterone concentrations than controls (p < 0.0001 for both groups; Fig. 2C; [Two-way RM-ANOVA: *Treatment effect*: *F*(1,15) = 203.44, *p* < 0.0001]). These results show that treatment with MDMA caused a significant increase in serum corticosterone levels 2 and 4 h post injection.

Experiment 2: Timely paired MDMA treatment with memory reactivation is necessary for effectiveness of treatment: We then tested the potential anxiolytic-like effect of MDMA on the underlying reconsolidation process. Rats were exposed to PSS and seven days thereafter, MDMA (5 mg/kg) or saline were administered with or without a trauma-cue. In addition, 2 groups of rats were exposed to PSS and six days thereafter, MDMA or saline were administered, one day before exposure to the situational reminder (i.e., MDMA treatment was unpaired from the reminder).

### Locomotor activity

**Fig. 3:**
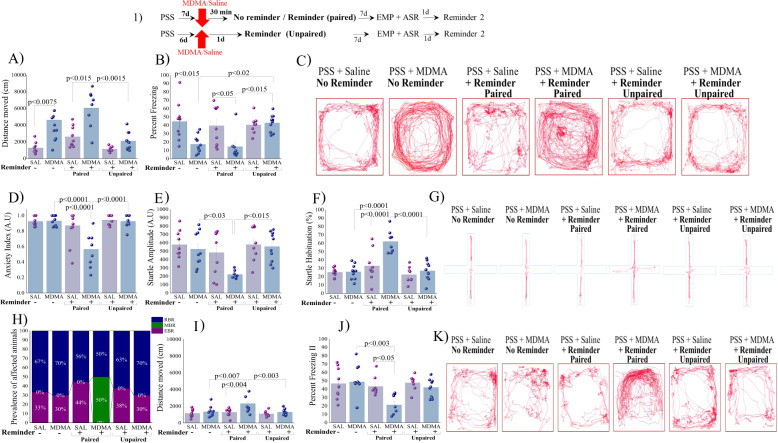
MDMA treatment timely paired with trauma-cue attenuates behavioral stress responses (1) Top panel: experimental protocol. Vertical arrows represent intraperitoneal MDMA (5 mg/kg) or saline injection. Rats were exposed for 10 min to predator-scent stress (PSS) on day 0. On day 7, rats received MDMA or saline and 30 min later were exposed for 10 min to a reminder (PSS + saline + Reminder: *n* = 9; PSS + MDMA + reminder (Paired): *n* = 8) or not (without reminder) (PSS + saline alone: *n* = 9; PSS + MDMA alone: *n* = 10). In addition, 2 groups of rats were exposed to PSS and six days thereafter, MDMA or saline were administered, one day before exposure to the situational reminder (PSS + saline + Reminder: *n* = 8; PSS + MDMA + Reminder (unpaired): *n* = 10). During this time **A** LMA (distance moved; measured in cm); **B** Percent freezing response [Two-way ANOVA: Treatment effect: (*F*(1,32) = 16.75, *p* < 0.0003)]; **C** Representative patterns of locomotor activity (cumulated values) in all groups. Behavioral measurements (EPM and ASR) were performed on day 14 and freezing behavior on day 15. **D** Anxiety index, which integrates the measured EPM behavioral measures; **E** Startle amplitude in the ASR paradigm; **F** Percentage of startle habituation in the ASR paradigm. **G** Representative accumulated movement track during a trial. **H** Prevalence of extreme behavioral response (EBR) vs. partial behavioral response (PBR) and minimal behavioral response (MBR) rats (in percentages). Significant differences were found between groups in the prevalence rates of individuals displaying an extreme, minimal, and partial behavioral response (Pearson *χ*^2^ = 17.62, df = 6, *p* < 0.0075). Finally, the effect of trauma-cue at day 15 on LMA (**I**) (distance moved; measured in cm; and **J** Percent freezing response. **K** Representative patterns of locomotor activity (cumulated values) in all groups. The experiments described below were performed with three different cohorts of animals; 19 rats were run in one experimental design, 17 rats in another experimental design and 18 rats in a third experimental design. Bars represent group means ± SEM and percentages.

MDMA treatment (with and without a reminder) significantly increased the LMA as compared to saline controls (*p* < 0.015 and *p* < 0.0075, respectively; Fig. 3A–C). MDMA treatment paired with a trauma-cue produced a significant increase in LMA compared to MDMA treatment which was unpaired with a trauma-cue (*p* < 0.0015; [Two-way ANOVA: *Treatment effect*: *F*(1,48) = 23.6, *p* < 0.0001; *Reminder effect:* *F*(2,48) = 8.4, *p* < 0.0008; *Treatment-Reminder interaction* = NS]). Accordingly, MDMA treatment (with and without a reminder) significantly decreased freezing behavior as compared to saline controls (*p* < 0.05 and *p* < 0.015, respectively; Fig. 3B). MDMA treatment unpaired with a trauma-cue produced significantly more freezing behavior compared to MDMA treatment which was paired with a trauma-cue (*p* < 0.015) or when given without reminder (*p* < 0.02); [Two-way ANOVA: *Treatment effect*: *F*(1,48) = 13.2, *p* < 0.0007; *Reminder effect*: *F*(2,48) = 3.63, *p* < 0.035; *Treatment-Reminder interaction*: *F*(2,48) = 4.34, *p* < 0.02]. *Elevated plus maze:* MDMA treatment paired with a trauma-cue significantly reduced the anxiety-index produced by prior PSS as compared to MDMA treatment unpaired with a trauma-cue (*p* < 0.0001), their control counterparts (saline+reminder) (*p* < 0.0001) and to MDMA treatment alone (without reminder) (*p* < 0.0001), Fig. 3D–G; [Two-way ANOVA: *Treatment effect*: *F*(1,48) = 10.42, *p* < 0.0025; *Reminder effect*: *F*(2,48) = 18.25, *p* < 0.0001; *Treatment-Reminder interaction:* *F*(2,48) = 9.2, *p* < 0.00045. Please see Supplementary Information 3.0 for all EPM parameters.

### Acoustic startle response

MDMA treatment paired with a trauma-cue significantly reduced the startle amplitude (Fig. 3E) and increased startle habituation (Fig. 3F) produced by prior PSS as compared to MDMA treatment unpaired with a trauma-cue (*p* < 0.015 and *p* < 0.0001, respectively), and to MDMA treatment alone (without reminder) (*p* < 0.03 and *p* < 0.0001, respectively). MDMA treatment paired with a trauma-cue significantly increased startle habituation than their control counterparts (saline+reminder) (*p* < 0.0001; [Two-way ANOVA: Startle response: *Treatment effect:* *F*(1,48) = 4.54, *p* < 0.04; *Reminder effect:* *F*(2,48) = 6.6, *p* < 0.003; *Treatment-Reminder interaction*: NS; Startle habituation: *Treatment effect*: *F*(1,48) = 12.38, *p* < 0.001; *Reminder effect*: *F*(2,48) = 20.68, *p* < 0.0001; *Treatment Reminder interaction*: *F*(2,48) = 7.5, *p* < 0.0015]). Taken together, MDMA treatment alone or unpaired to trauma-sue showed no efficacy in the EPM and ASR paradigms.

### Relative prevalence rates of behavioral response, according to CBC

There were significant differences in the prevalence rates of MBR among groups (Pearson *χ*^2^ = 24.84, df = 5, *p* < 0.002; Fig. 3H). The prevalence of MBR among exposed rats treated with MDMA paired with a trauma-cue was 50.0% of the total population and differed significantly from their control counterparts (saline + reminder) (*χ*^2^ = 5.83, *p* < 0.01), the PSS-exposed group treated with MDMA alone (*χ*^2^ = 6.43, *p* < 0.015) or MDMA treatment unpaired with a trauma-cue (*χ*^2^ = 6.43, *p* < 0.015), where there were no MBR cases. There were no significant differences in EBR or PBR prevalence among groups. These analyses indicate that memory reactivation is necessary for MDMA treatment to be effective.

Freezing behavior at Day 15*:* MDMA treatment paired with a trauma-cue significantly increased the distance moved compared to MDMA treatment unpaired with a trauma-cue (*p* < 0.003), to their control counterparts (saline+reminder) (*p* < 0.004) or to MDMA treatment alone (without trauma-cue) (*p* < 0.007, Fig. 3I–K; [Two-way ANOVA: *PSS-exposure effect*: *F*(1,48) = 10.2, *p* < 0.003; Treatment effect: *F*(2,48) = 6.7, *p* < 0.003; *Exposure-treatment interaction effects:* *F*(2,48) = 3.6, *p* < 0.04]). Accordingly, MDMA treatment paired with a trauma-cue significantly reduced immobility than saline treatment (*p* < 0.05), or MDMA treatment alone (*p* < 0.003, Fig. 3J; [Two-way ANOVA: *PSS-exposure effect:* *F*(1,48) = 5.2, *p* < 0.03; Treatment effect: *F*(2,48) = 5.7, *p* < 0.0065; *Exposure-treatment interaction effects*: *F*(2,48) = 3.2, *p* < 0.05)].

Experiment 3: In Lewis rats, MDMA treatment has no long-term behavioral effects: We hypothesized that the mechanism by which MDMA attenuates stress-related behavior after exposure to PSS involves glucocorticoids. To test this hypothesis, we first tested the effects of MDMA/saline in Lewis rats with blunted HPA-response.

### Locomotor activity

**Fig. 4:**
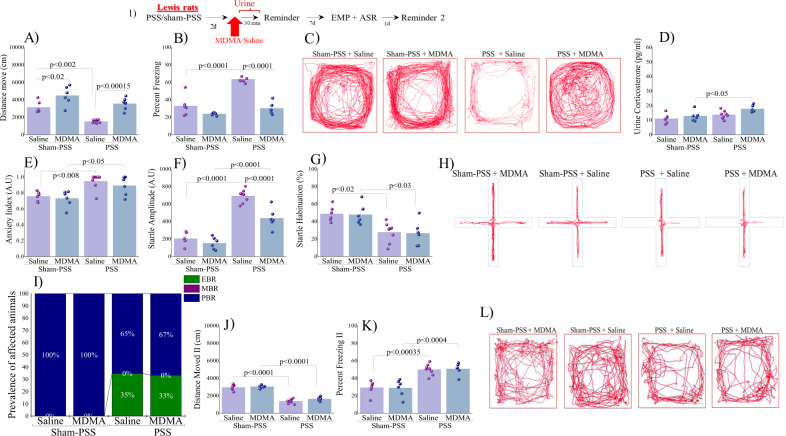
In Lewis rats, MDMA treatment has no long-term behavioral effects (1) Top panel: experimental protocol. Vertical arrows represent intraperitoneal MDMA (5 mg/kg) or saline injection. Lewis rats were exposed for 10 min to predator-scent stress (PSS) or to sham-PSS on day 0. On day 7, rats received MDMA (sham-PSS + MDMA: *n* = 6; PSS-exposed + MDMA: *n* = 6) or saline (sham-PSS + saline: *n* = 6; PSS-exposed + saline: *n* = 8) and 30 min later were exposed to a trauma-cue for 10 min. During this time **A** LMA (distance moved; measured in cm); **B** Percent freezing response; **C** Representative patterns of locomotor activity (cumulated values) in all groups. **D** Urine corticosterone concentrations (pg/ml) were measured. Behavioral measurements (EPM and ASR) were performed on day 14 and freezing behavior on day 15. **E** Anxiety index, which integrates the measured EPM behavioral measures; **F** Startle amplitude in the ASR paradigm; **G** Percentage of startle habituation in the ASR paradigm. **H** Representative accumulated movement track during a trial. **I** Prevalence of extreme behavioral response (EBR) vs. partial behavioral response (PBR) and minimal behavioral response (MBR) rats (in percentages). Finally, the effect of trauma-cue at day 15 on LMA **J** (distance moved; measured in cm); and **K** Percent freezing response; **L** Representative patterns of locomotor activity (cumulated values) in all groups. The experiments described below were performed with two different cohorts of animals; 13 rats were run in one experimental design, and 13 rats in another experimental design. Bars represent group means ± SEM and percentages.

In Lewis rats MDMA treatment (sham-PSS and PSS-exposure) significantly increased the LMA as compared to their saline controls (*p* < 0.02 and *p* < 0.00015, respectively, Fig. 4A–C; [Two-way ANOVA: *PSS-exposure effect*: *F*(1,22) = 21.6, *p* < 0.0002; *Treatment effect:* *F*(1,22) = 37.7, *p* < 0.0001; *Exposure-Treatment interaction:* NS]).

The total distance moved was significantly lower in exposed group treated with saline than sham-PSS control group (p < 0.002). Accordingly, MDMA treatment reduced the freezing behavior (immobility) induced by prior PSS as compared to exposed rats treated with saline (*p* < 0.0001, Fig. 4B, C; [Two-way ANOVA: *PSS-exposure effect*: *F*(1,22) = 46.9, *p* < 0.0001; *Treatment effect:* *F*(1,22) = 61.76, *p* < 0.0001; *Exposure-Treatment interaction*: *F*(1,22) = 20.1, *p* < 0.0002]). Taken together, in Lewis rats, MDMA treatment produced a significant hyper-locomotor response compared to saline controls.

Urine corticosterone: Lewis rats exposed to PSS and treated with MDMA displayed significantly higher urine corticosterone concentrations than sham-PSS control (*p* < 0.05, Fig. 4D; [Two-way ANOVA: *PSS-exposure effect*: *F*(1,22) = 10.4, *p* < 0.004; *Treatment effect:* *F*(1,22) = 6.1, *p* < 0.025; *Exposure-Treatment interaction*: NS]).

### Behavioral stress responses:

#### Elevated plus maze

Exposed groups treated with saline or MDMA showed a significantly increased anxiety-index as compared to unexposed controls treated with saline (*p* < 0.008 and *p* < 0.05, respectively; Fig. 4E; [Two-way ANOVA: *PSS-exposure effect*: *F*(1,22) = 21.5, *p* < 0.00015; *Treatment effect:* NS; *Exposure-Treatment interaction:* NS]). Please see Supplementary Information 4.0 for all parameters.

#### Acoustic startle response

Exposed groups had significantly increased startle amplitude and decreased habituation compared to unexposed controls (saline: *p* < 0.0001 and *p* < 0.02, and MDMA: *p* < 0.0001 and *p* < 0.035, respectively; Fig. 4F–H). Exposed rats treated with MDMA showed a significant decrease in startle amplitude compared to exposed rats treated with saline (*p* < 0.0001; [Two-way ANOVA: Startle amplitude: *PSS-exposure effect*: *F*(1,22) = 136.2, *p* < 0.0001; *Treatment effect:* *F*(1,22) = 21.2, *p* < 0.00015; *Exposure–Treatment interaction*: *F*(1,22) = 9.3, p < 0.006; Startle habituation: *PSS-exposure effect*: *F*(1,22) = 21.3, *p* < 0.00015; *Treatment effect:* NS; *Exposure-Treatment interaction*: NS]).

#### CBC model classification

No significant differences were found in the prevalence of EBR, PBR and MBR between groups (Fig. 4I). EBR prevalence was lower in sham-exposed Lewis rats (0%) than in exposed rats treated with saline (2/6, 33.33%) or MDMA (2/6, 33.33%).

#### Freezing behavior at day 15

PSS-exposed rats treated with saline or MDMA exhibited a significant decrease in the distance moved in the arena as compared to sham-PSS rats treated with saline (*p* < 0.0001 for both groups, Fig. 4J–L; [Two-way ANOVA: *PSSexposure effect:* *F*(1,22) = 239.6, *p* < 0.0001; *Treatment effect:* NS, (*F*(1,22) = 3.2, *p* = 0.09); *Exposure-Treatment interaction*: NS]). Accordingly, exposed rats treated with saline or MDMA displayed significantly less immobility than sham-exposed rats (*p* < 0.000355 and *p* < 0.0004, respectively; Fig. 4K, L; [Two-way ANOVA: *PSS-exposure effect:* *F*(1,22) = 49.1, *p* < 0.0001; *Treatment effect*: NS; *Exposure-Treatment interaction*: NS]). In Lewis rats, therefore, MDMA treatment has no long-term behavioral effects.

Experiment 4: The glucocorticoid receptor and the serotonin receptors 5-HT-_1A_ and 5-HT-_2A_ are necessary for the anxiolytic effects of MDMA treatment: To test the extent to which the observed anxiolytic effects of MDMA are directly associated with the GRs, or the serotonin receptors (5-HT1A, 5-HT2A and 5-HT2C), we injected rats with either RU486, Ketanserin, Pindolol and SB242084 or saline, with MDMA treatment.

#### Locomotor activity

**Fig. 5:**
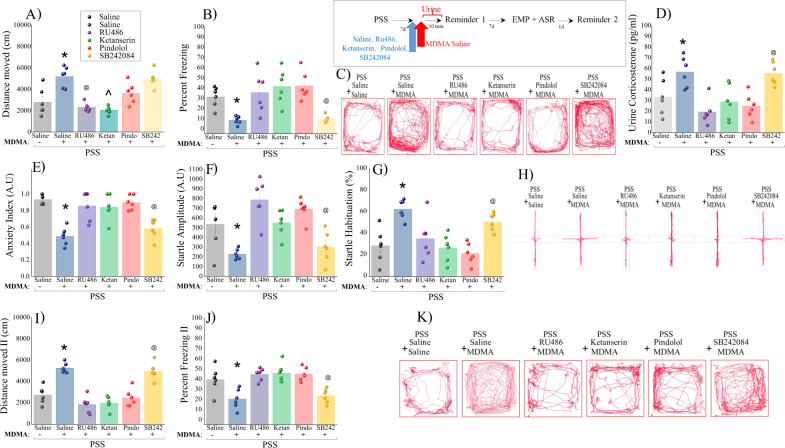
The glucocorticoid receptor and the serotonin receptors 5-HT-_1A_ and 5-HT-_2A_ are necessary for the anxiolytic effects of MDMA: (1) Top panel: experimental protocol. Rats were exposed for 10 min to predator-scent stress (PSS) on day 0. On day 7 rats were intraperitoneally injected with either saline, Ru486 (7.5 mg) (GR antagonist mifepristone), Ketanserin (5 mg/kg) (5-HT_2A_ R antagonist), Pindolol (0.3 mg/kg) (5-HT_1A_ R antagonist) or SB242084 (0.3 mg/kg) (5-HT_2C_ R antagonist) and 30 min before MDMA (5 mg/kg|) or saline injection, administered 30 min before a brief memory reactivation session. There were 6 rats per group. During this time **A** LMA (distance moved; measured in cm); **B** Percent freezing response; **C** Representative patterns of locomotor activity (cumulated values) in all groups. **D** Urine corticosterone concentrations** (pg/ml). Behavioral measurements (EPM and ASR) were performed on day 14 and freezing behavior on day 15. **E** Anxiety index, which integrates the measured EPM behavioral measures; **F** Startle amplitude in the ASR paradigm; **G** Percentage of startle habituation in the ASR paradigm. **H** Representative accumulated movement track of the rats during a trial. Finally, the effect of trauma-cue at day 15 on LMA **I** (distance moved; measured in cm); and **J** Percent freezing response; **K** Representative patterns of locomotor activity (cumulated values) in all groups. The experiments described below were performed with two different cohorts of animals; 18 rats were run in one experimental design, and 18 rats in another experimental design. Bars represent group means ± SEM and percentages.

Pretreatment with saline before MDMA increased the distance moved in the arena (Fig. 5A–C) and decreased freezing responses (Fig. 5B, C) as compared to saline+saline treatment group (*p* < 0.0005, *p* < 0.05, respectively) and to groups pre-treated with RU486, Ketanserin, or Pindolol (Distance moved: *p* < 0.001, *p* < 0.001 and *p* < 0.05. Percent freezing: *p* < 0.02, *p* < 0.002 and *p* < 0.0015, respectively). Pre-treatment with SB242084 before MDMA increased LMA and decreased freezing as compared to groups pretreated with RU486, Ketanserin, or Pindolol (Distance moved: *p* < 0.00015, *p* < 0.045. % freezing: *p* < 0.025, *p* < 0.003 and *p* < 0.0025, respectively; [One-way ANOVA: Distance moved: *F*(5,30) = 14.87, *p* < 0.0001; Percent freezing: *F*(5,30) = 8.4, *p* < 0.0001]).

#### Urine corticosterone

Pretreatment with saline or SB242084 before MDMA treatment significantly increased corticosterone concentrations compared to groups pretreated with RU486, Ketanserin, or Pindolol (saline-MDMA: *p* < 0.0009, *p* < 0.025 and *p* < 0.006).

SB242084 + MDMA: *p* < 0.0015, *p* < 0.035 and *p* < 0.0069, respectively; Fig. 5D; [One-way ANOVA: *F*(5,30) = 8.0, *p* < 0.0001].

#### GR, 5-HT2 R, and 5-HT1A R are required for MDMA’s anxiolytic effects

To test the extent to which the observed anxiolytic effects of MDMA are directly associated with GR, 5HT_2A_, 5-HT_1A_ or 5-HT_2C_, we injected one of the following, along with MDMA:

Glucocorticoid receptor antagonist (RA) Ru496, 5-HT_2A_ RA Ketanserin, 5-HT_1A_ RA Pindolol and 5-HT_2C_ RA SB24084 or saline. All antagonists were injected 60 min before MDMA, which was injected 30 min before the trauma-cue, and 7 days after PSS. Rat behavior was evaluated in the EPM and ASR paradigms 14 days after PSS exposure. Since no significant differences were observed in the behavioral tests (EMP and ASR) between the saline-control group and the groups that were exposed to sham-PSS and pre-treated with Ru486 (*N* = 3), Ketanserin (*N* = 3), Pindolol (*N* = 4), and SB242084 (*N* = 3) prior to MDMA treatment, we present only the results for actual PSS exposure with the pre-treatment.

In the *elevated plus maze*, rats exposed to PSS and pretreated with RU486, Ketanserin, or Pindolol before MDMA treatment exhibited a significantly higher anxiety index than the saline-treated groups (*p* < 0.0008, *p* < 0.002, *p* < 0.00035, respectively; Fig. 5E–H; [One-way ANOVA: *F*(5,30) = 13.4, *p* < 0.0001]). Therefore, pretreatment with RU486, Ketanserin, or Pindolol completely prevented the anxiolytic-like effects of MDMA in rats exposed to PSS.

Please see Supplementary Information 5.0 for all parameters.

In the *startle response paradigm*, rats exposed to PSS and pre-treated with RU486, Ketanserin, or Pindolol before MDMA treatment had significantly increased startle amplitude (*p* < 0.0003, *p* < 0.0004, *p* < 0.0006, respectively; Fig. 5F) and exhibited a significantly lower startle habituation than saline-treated MDMA rats (*p* < 0.0001, *p* < 0.025, *p* < 0.0006, respectively; Fig. 5G; [One-way ANOVA: Startle amplitude: *F*(5,30) = 10.4, *p* < 0.0001; Startle habituation: *F*(5,30) = 8.7, *p* < 0.0001]).

Freezing behavior at Day 15: Rats pretreated with saline or SB242084 before MDMA treatment showed a significant increase in the distance moved in the arena as compared to rats pretreated with RU486, Ketanserin, or Pindolol before MDMA (*p* < 0.0001 for all, Fig. 5I–K; [One-way ANOVA: *F*(5,30) = 25.8, *p* < 0.0001]). Accordingly, rats pretreated with RU486, Ketanserin, or Pindolol before MDMA treatment displayed significantly more immobility than exposed rats treated with saline + MDMA (*p* < 0.0001) or pretreated with SB242084 RA (*p* < 0.0001, Fig. 5J, K; [One-way ANOVA: *F*(5,30) = 9.98, *p* < 0.0001]).

## Discussion

The main findings of this study demonstrate that MDMA injection 30 min before exposure to a trauma-cue (memory reactivation) 7 days after PSS-exposure has observable long-term effects on stress-induced behavior in a translational PTSD model. Single-dose MDMA (5 mg/kg) resulted in a significant reduction of anxiety-related behavior and attenuation of PTSD-related responses compared to saline treatment (8 days later). Retrospective data analysis revealed that the MDMA regimen reduced the prevalence rates of PTSD phenotypes (EBR) to nil, with a concomitant increase in MBR (unaffected) prevalence to 44% as compared to saline treatment (MBR = 0%), (i.e., a significant overall shift towards more adaptive behavioral response patterns to PSS). The MDMA-treated group also demonstrated markedly less extreme freezing responses to re-exposure to the trauma-cue than the saline-control group (27.7 5% vs. 47.5%, respectively). Freezing behavior indicates a sense of immediate threat and intense fear. For a neutral stimulus to cause a freezing response, (i.e., to act as a trauma-cue), it requires the involvement of contextual association with the stressor through memory-related processes. The finding that MDMA administration prior to such exposure effectively prevented this effect raises the possibility that it affects memory-related processes.

Importantly, MDMA had no long-term intrinsic pharmacotherapeutic efficacy in and of itself and was effective in attenuating behavioral responses only when paired with trauma reactivation by a trauma-cue (i.e., 30 min before) (suggesting that the memory reactivation is necessary), that is, animals who actively “recalled” their stressful event under the influence of MDMA subsequently exhibited lower rates of severely affected individuals compared to either MDMA treatment alone or to memory reactivation with saline administration. As memory reactivation may initiate two different processes, reconsolidation and extinction [39], it is unclear whether MDMA modifies one or the other (or both).

Recent studies have shown that the duration of a reminder event may be an important determinant of subsequent memory processing: brief reminders lead to reconsolidation, whereas prolonged reminders induce extinction [40–44]. Furthermore, Bustos et al. [40] reported that memories become increasingly resistant to disruption with age. Older memories are less susceptible to interference than newer or recently acquired ones [39, 40, 45] and longer duration of reminders are necessary to destabilize older memories. Similarly, longer reminders were required to induce reconsolidation of stronger memories [40]. Considering the duration of the memory reactivation (relatively brief), the age of original trauma memory (7 days), and the time span between reactivation and behavioral testing (7 days) [39, 40], one plausible explanation for our data is that MDMA injection paired with memory reactivation may modify, update or impair the original traumatic memory trace through the reconsolidation process (and thus reduce stress-related behavioral responses).

In line with our results, Hake et al. [34] reported that MDMA interferes with the reconsolidation of both cued and contextual fear memory. When administered immediately after a brief fear memory retrieval session, MDMA reduced subsequent expression of fear to the conditioned cue or context [34]. This data is consistent with a memory-impairing effect of MDMA and implicate as the likely mechanism underlying the long-lasting therapeutic effects of MDMA-assisted psychotherapy [34] interference with fear memory reconsolidation, rather than an enhancement of fear extinction. In contrast, Young et al. [46] report that, in mice, MDMA administered prior to cued fear extinction enhances fear extinction memory recall and reduces fear renewal induces spontaneous recovery. However, it is important to note that there is an interaction between the extinction and reconsolidation processes [39] and we cannot exclude that the MDMA may act on both processes [39, 47, 48]. Similar conclusions have been reported in clinical trials in humans [47, 49].

Interestingly, the beneficial behavioral effects of MDMA treatment paired with a trauma-cue were accompanied by a normalization of the dendritic cytoarchitecture of DG and BLA neurons. Whilst exposed rats treated with saline displayed extensive neuronal retraction in the DG and proliferation in the BLA, MDMA treatment was associated with a dramatic increase in the dendritic arborization of DG granular neurons, and a decrease in that of BLA pyramidal neurons. MDMA alone did not alter the dendritic cytoarchitecture.

Consistent with previous studies [22, 50], we found that MDMA treatment produces a significant elevation of circulating corticosterone 2 h post injection, which remains for over 4 h. As corticosterone is a potent modulator of the memory circuits engaged in reprocessing of fear and traumatic memories [49], and as cortisol has been shown to enhance emotional memory consolidation process [51, 52], we assumed that the pharmacological corticosterone elevations during memory reactivation resulting from MDMA treatment is actively involved in MDMA-induced anxiolytic-like behavioral responses.

To test this assumption, we first evaluated the effects of a single MDMA dose on behavioral stress responses in inbred Lewis rats and subsequently, manipulated GR expression (in Sprague-Dawley rats) by either administering a direct antagonist (RU486) or indirect antagonists, and through blockade of 5-HT_2_, 5-HT_1A_, in a second set of experiments. We found that absence of GR reactivity, whether due to genetics (Lewis rats) or pharmacological blockade (RU486) prevents the above MDMA-induced reduction in anxiety-like behavioral responses. In Lewis rats MDMA treatment to was unable to reverse the behavioral and physiological changes induced by PSS. This was the case both in the EPM and the ASR tests. Consistently, using CBC no differences were observed among the prevalences of the three responder types. However, a significant decrease in ASR amplitude was noted with the MDMA treatment in Lewis rats compared to saline, although the startle amplitude was significantly higher than in sham-exposed control rats. The only statistically significant effect of MDMA in Lewis rats was an increase in locomotor activity a and a corresponding decrease in freezing responses during memory reactivation compared with the PSS saline group, similar to the responses observed in Sprague-Dawley rats. Moreover, in Lewis rats MDMA treatment administered adjunctively to traumatic memory reactivation did not increase in corticosterone concentration. This study suggests that the HPA axis plays an important role in mediating MDMA-induced anxiolytic-like behavioral responses. In addition, pharmacologically blocking GR reactivity by RU486, yielded similar results; MDMA treatment paired with a trauma-cue did not trigger an increase in corticosterone concentration and caused elevated anxiety-like responses. We also found that pretreatment with Ketanserin (5-HT_2A_-receptor antagonists), or Pindolol (5-HT_1A_-receptor antagonists) significantly prevented MDMA-induced hyper-corticosterone levels and prevented the reduction in anxiety-related behavior after PSS exposure. On the other hand, pretreatment with SB242084 (a 5-HT_2C_-receptor antagonist) did not prevent MDMA from inducing these behavioral changes. Because activation of either 5-HT_2A_ or 5-HT_1A_ receptors results in an elevation in corticosterone concentrations [53], MDMA-induced elevated corticosterone concentrations are not only due to direct release but result from an indirect serotonergic influence. Moreover, the HPA axis is also involved in the indirect effects of MDMA treatment by increasing locomotor activity, which peripherally and centrally activate the HPA axis, and lead to a big increase in corticosterone concentration [54–57]. In our animal model, both the HPA axis and 5-HT systems seem to be involved in the anxiolytic-like effects of MDMA.

As cortisol has been shown to enhance emotional memory consolidation process [51, 52, 58], and based on the similarity between memory reconsolidation and initial consolidation, we assume that the pharmacological corticosterone elevations during memory reactivation resulting from MDMA treatment, would lead to enhancing reconsolidation of the reactivated trauma memories as well. However, the effects of GCs on reconsolidation of fear memory are not clear as receptor antagonists [59] and agonists [60, 61] have both been reported to impair reactivated memories of different types, which suggest both impairing and enhancing effects [58]. However, based on an inverted U-shaped dose-response relationship between corticosterone and memory processes, high levels of corticosterone elicited by MDMA treatment could impair memory reconsolidation, thereby reducing the trauma memory. Future studies are required to explore the specific mechanisms of this effect.

## Conclusions

A single administration of MDMA paired with a trauma-cue 7 days after exposure to a psychogenic stressor significantly reduced the prevalence of a PTSD-phenotype 14 days later, improved resilience to a trauma cue on day 15, and normalized the cytoarchitectural changes in dendritic complexity induced by PSS. In the absence of memory reactivation, MDMA failed to alter behavioral stress responses.

Overall, the findings of this study parallel clinical data from MDMA-assistant trauma therapy. Specifically, MDMA demonstrated no intrinsic “therapeutic” value, unless administered shortly prior to induced-triggering of trauma memories, bringing about an attenuation of subsequent behavioral sequelae, compared to untreated animals exposed to the trauma-memory triggering conditions.

It is plausible that MDMA treatment paired with memory reactivation acts, both directly and indirectly, on the HPA axis and on serotonergic transmission to modulate, modify or update the original traumatic memory trace through reconsolidation processes thus reducing stress-related sequelae. This pharmacological profile of MDMA may provide a new direction for future drug development for patients with treatment-resistant PTSD.

## Supplementary information

Supplementary Materials 1

Supplementary Materials 2

Supplementary Materials 3

Supplementary Materials 4

Supplementary Materials 5
