# Supplementary material for: MDMA treatment paired with a trauma-cue promotes adaptive stress responses in a translational model of PTSD in rats
Source: Transl Psychiatry. 2022 May 3;12:181. doi: 10.1038/s41398-022-01952-8 (PMC9064970; doi:10.1038/s41398-022-01952-8)
Supplement: Supplementary file 1 — Supplementary Materials 1 [file 41398_2022_1952_MOESM1_ESM.docx]

**Supplementary Materials #1**

1.1 Behavioral Assessments

All behavioral tests were performed in a closed, quiet, light-controlled room between 10:00-16:00 hr. All behavioral tests were video-recorded for future analysis using the ETHO-VISION program (Noldus Information Technology, Wageningen, The Netherlands) by an investigator blinded to the experimental protocol.

***The elevated plus-maze:*** The maze is a plus-shaped platform with two opposing open and two opposing closed arms (surrounded by 41 cm high opaque walls on three sides) [138]. Rats were placed on the central platform facing an open arm and allowed to explore the maze for 5 min. Each test was videotaped and subsequently scored by an independent observer. An arm entry was defined as entering an arm with all four paws.

Behaviors assessed were: time spent (duration) in open and closed arms on the central platform; the number of open and closed arm entries; and total exploration (entries into all arms). Total exploration was calculated as the number of entries into an arm of the maze in order to distinguish between impaired exploratory behavior, exploration limited to closed arms (avoidance), and free exploration. "Anxiety Index", an index that integrates the elevated plus maze behavioral measures, was calculated as follows:

$$\begin{matrix} Anxiety & Index=1-\left[\frac{\left(\frac{\begin{matrix} \begin{matrix} \begin{matrix} time & spent \end{matrix} & in & the \end{matrix} & open & arms \end{matrix}}{\begin{matrix} \begin{matrix} total & time & on \end{matrix} & the & maze \end{matrix}}\right)+}{\begin{matrix} & \begin{matrix} \begin{matrix} \begin{matrix} \begin{matrix} \begin{matrix} \begin{matrix} & & \end{matrix} & & \end{matrix} & & \end{matrix} & & \end{matrix} & & \end{matrix} & 2 \end{matrix} \end{matrix}}\frac{\left(\frac{\begin{matrix} \begin{matrix} \begin{matrix} number & of \end{matrix} & entries & to \end{matrix} & the & \begin{matrix} open & arms \end{matrix} \end{matrix}}{\begin{matrix} \begin{matrix} total & \exp loration & on \end{matrix} & the & maze \end{matrix}}\right)}{}\right] \end{matrix}$$

Anxiety Index values range from 0-1 where an increase in the index expresses increased anxiety-like behavior [23], [139]

***Acoustic startle response:*** Startle response was measured using two ventilated startle chambers (SR-LAB system, San Diego Instruments, San Diego, CA). The SR-LAB calibration unit was used routinely to ensure consistent stabilimeter sensitivity between test chambers and over time. Each Plexiglas cylinder rests on a platform inside a sound-proofed, ventilated chamber. Any movement inside the tube is detected by a piezoelectric accelerometer below the frame. Sound levels within each test chamber are measured routinely using a sound level meter (Radio Shack) to ensure consistent presentation. Each test session started with a 5-min acclimatization period to background white noise of 68 dB, following by 30 acoustic startle trial stimuli in 6 blocks (110 dB white noise of 40 ms duration with 30 or 45 s inter-trial interval). The behavioral assessment consisted of the mean startle amplitude (averaged over all 30 trials) and the percent of startle habituation to repeated presentation of the acoustic pulse. Percent habituation -- the percent change between the response to the first block of sound stimuli and the last – was calculated as follows:

1.2. The Cut-off Behavioral Criteria Model of PTSD

The behavioral responses of animals in both the elevated plus maze and acoustic startle response tests were first analyzed by group (e.g., blast-exposed vs. sham-exposed vs. unexposed). Subsequently, individual animals were classified according to their behavioral response pattern on both the elevated plus maze and acoustic startle response, by using the cut-off behavioral criteria model, as exhibiting either “extreme behavioral response” (EBR) or “minimal behavioral response” (MBR) (1-7). Those that fulfilled neither set of criteria were labeled, exhibiting a “partial behavioral response” (PBR). This procedure is detailed in Figure S1.

**
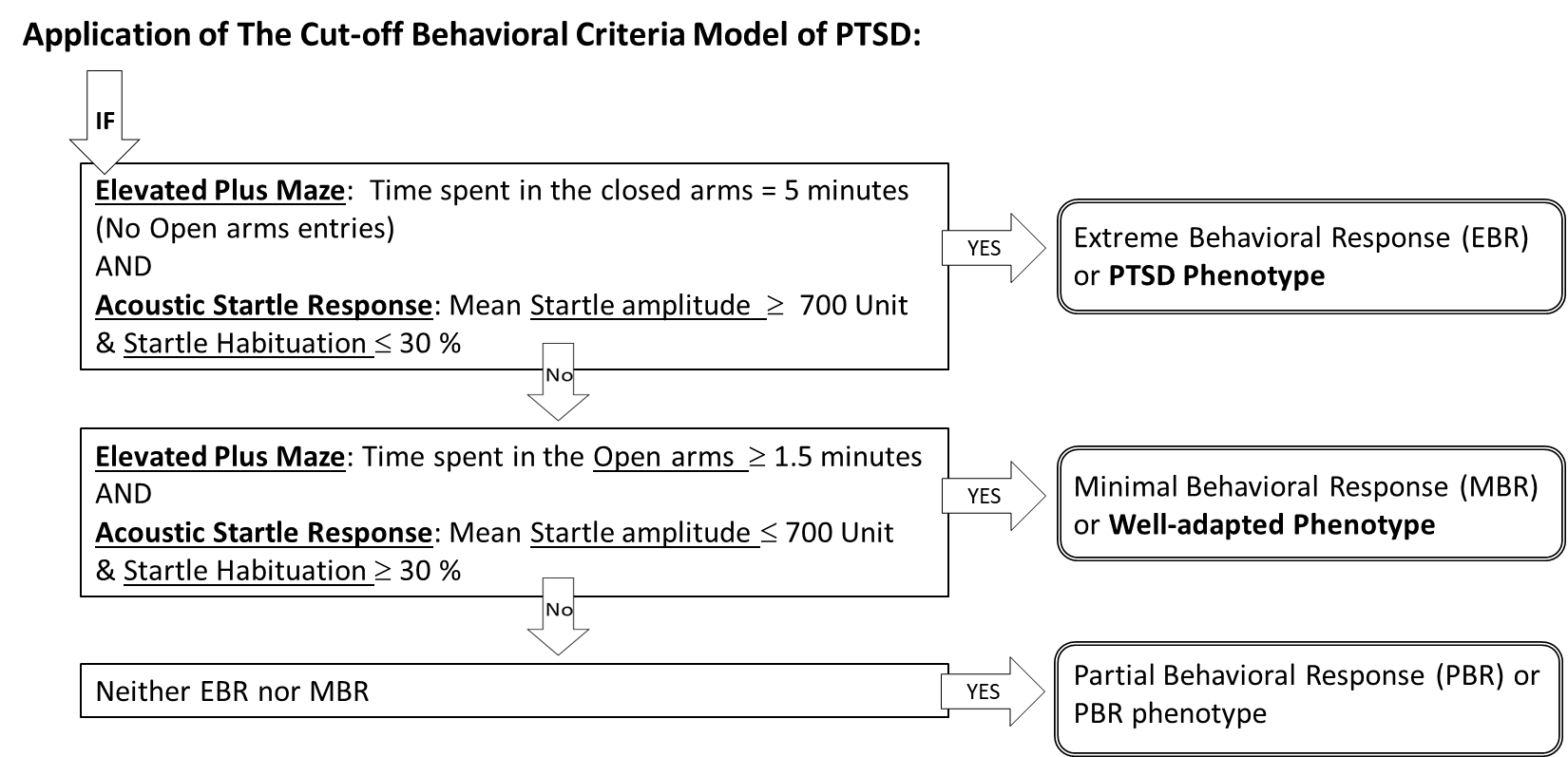
**

**Figure S1.** The cut-off behavioral criteria algorithm: To approximate the behavioral model to contemporary clinical conceptions of post-traumatic stress disorder (PTSD), we classified animals into groups according to the degree of response to the stressor (PSS, predator scent stressor), i.e., the degree to which the behavior of an individual is altered or disrupted. To this end, behavioral criteria were defined and then complemented by the definition of cut-off criteria, which reflect the severity of response; this parallels inclusion and exclusion criteria applied in clinical research. The procedure requires the following steps: (**A**) Verification of global effect: the data must demonstrate that the stressor has a significant effect on the overall behavior of PSS- versus Sham PSS-exposed populations at the time of assessment; (**B**) Application of the cut-off behavioral criteria to the data: to maximize the resolution and minimize false positives, extreme responses to the stress in both the elevated plus-maze and acoustic startle response paradigms (performed sequentially) are required for “inclusion” into the extreme behavioral response (EBR) group. A negligible response in both paradigms is required for inclusion into the minimal behavioral response (MBR) group. Individuals that are not classified as having an EBR or an MBR are, by default, classified as having a partial behavioral response (PBR).

**1.3 Golgi-Cox Staining**

Brains were rinsed with distilled water and immersed in impregnation solution containing potassium dichromate, mercuric chloride and potassium chromate. Brains were left undisturbed in the dark for 2.5 weeks. After the 2.5 weeks, brains were immersed in 30% sucrose at 4 °C. Two to four days later coronal sections (100 μm) were cut using a cryostat (Microtome HM 500 OM cryostat, kept at −22 to −25 °C) in a bath of 15% sucrose, and the slices were stored in the dark at 4 °C in 15% sucrose solution until mounting. Sections were mounted on gelatin-coated slides and firmly pressed using moist filter paper to prevent the slices from falling off the slide during development ([Gibb and Kolb, 1998](http://www.sciencedirect.com/science/article/pii/S0306452212009797#b0075)). Slides were placed in a humidity chamber in the dark and were stored at 4°C overnight. For development, slides were rinsed with distilled water twice for 2 min and then placed in developing solutions provided in the FD GolgiStain Kit, dehydrated via a graded ethanol series (50%–100%, 4 min each rinse), cleared with xylene for 8 min, and coverslipped with Permount (Fisher Scientific, Netherlands).

**Neuronal reconstruction and morphometric analysis:** In order to obtain accurate measurements of dendritic parameters, strict criteria were adopted for the selection of the filled neurons before quantitative analysis: 1) Only well-impregnated neurons were chosen for the histological analysis. 2) Granule cells were included in this analysis only if the cell body and primary dendrites were clearly stained and easily distinguishable from those of neighboring cell bodies and their dendrites. 3) Granule cells were sampled from the suprapyramidal blades (SPB) of the DG, in both the right and left sides of the brain. 4) Granule cells from the inner granule zone (IGZ) were included in this analysis (because the dendritic morphology of hippocampal DG cells varies with their position in the granule cell layer (8)). A cell was classified as belonging to the IGZ if the entire soma was positioned in the inner half of the granule cells layer (GCL). Granule cells whose soma was intersected by the midline of the GCL, in the outer granule zone (OGZ), or in the subgranular zone were not included in any analysis.

We performed an analysis to characterize the extent that dendrites branched out from both somal and dendritic sites. Primary dendrites were defined as direct extensions from the soma of at least 10 µm in length. Only DGs with at least one primary dendrite >10 µm in total length were analyzed. When a primary dendrite bifurcated at a branch point, the dendrites extending from that branch point were classified as secondary dendrites. We extended this analysis to include tertiary (3), quaternary (4), quinary (5) and senary (6) order dendrites. This procedure provides an additional measure of the pattern of dendritic arborization, allowing a more comprehensive analysis of differences in the branch patterns of the dendrites themselves. We also performed a Sholl analysis (9). A series of concentric rings, spaced 25 μm apart, was placed over the neuron and centered on the cell body, and the number of dendrite crossings as a function of distance was recorded.

All slides were coded and the analysis was performed with the experimenter blind as to the origin of the slides. Dendritic morphology was observed by fluorescent microscopy (Leica, Germany). A 0.5 µm interval z-series was captured throughout the extent of the dendritic arbor with a CCD camera (Leica, Germany) controlled by LAS software.

**REFERENCES**

1. Cohen H, Zohar J (2004): An animal model of posttraumatic stress disorder: the use of cut-off behavioral criteria. *Ann N Y Acad Sci*. 1032:167-178.

2. Cohen H, Zohar J, Matar M (2003): The relevance of differential response to trauma in an animal model of posttraumatic stress disorder. *Biological psychiatry*. 53:463-473.

3. Cohen H, Zohar J, Matar MA, Kaplan Z, Geva AB (2005): Unsupervised fuzzy clustering analysis supports behavioral cutoff criteria in an animal model of posttraumatic stress disorder. *Biological psychiatry*. 58:640-650.

4. Cohen H, Zohar J, Matar MA, Zeev K, Loewenthal U, Richter-Levin G (2004): Setting apart the affected: the use of behavioral criteria in animal models of post traumatic stress disorder. *Neuropsychopharmacology : official publication of the American College of Neuropsychopharmacology*. 29:1962-1970.

5. Cohen H, Kozlovsky N, Alona C, Matar MA, Joseph Z (2012): Animal model for PTSD: from clinical concept to translational research. *Neuropharmacology*. 62:715-724.

6. Matar MA, Zohar J, Cohen H (2013): Translationally relevant modeling of PTSD in rodents. *Cell and tissue research*. 354:127-139.

7. Cohen H, Matar MA, Joseph Z (2013): Animal models of post-traumatic stress disorder. *Current protocols in neuroscience*. Chapter 9:Unit 9.45.

8. Green EJ, Juraska JM (1985): The dendritic morphology of hippocampal dentate granule cells varies with their position in the granule cell layer: a quantitative Golgi study. *Exp Brain Res*. 59:582-586.

9. Sholl DA (1956): The measurable parameters of the cerebral cortex and their significance in its organization. *Prog Neurobiol* 2:324-333.
