# Supplementary material for: MDMA treatment paired with a trauma-cue promotes adaptive stress responses in a translational model of PTSD in rats
Source: Transl Psychiatry. 2022 May 3;12:181. doi: 10.1038/s41398-022-01952-8 (PMC9064970; doi:10.1038/s41398-022-01952-8)
Supplement: Supplementary file 2 — Supplementary Materials 2 [file 41398_2022_1952_MOESM2_ESM.docx]

**Supplementary Materials #2**

**RESULTS:**

**2.1. Experiment 1: MDMA treatment attenuates behavioral stress responses:**

***
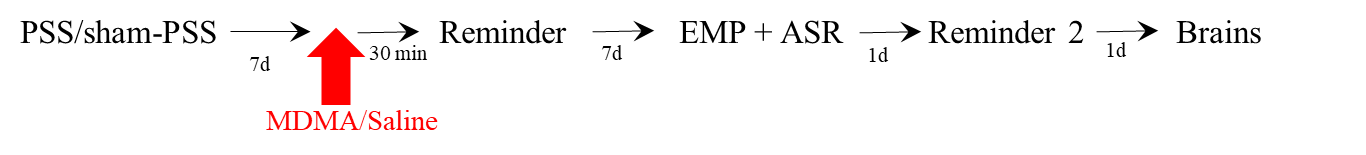
***

The behavioral procedure used for the unexposed and PSS-exposed rats. Vertical arrows represent intraperitoneal injection (MDMA (5 mg/kg) or Saline).

***Elevated plus Maze:*** All the rats were tested in the elevated plus maze (EPM) and acoustic startle response (ASR) paradigm on day 7. In term of time spent in open arms, two-way ANOVA revealed a significant effect of PSS-exposure (F(1,36)=4.0, p<0.05), and a significant PSS exposure-treatment interaction effect (F(1,36)=12.8, p<0.0015) (Figure S1A). No effect was observed for treatment. Post-hoc Bonferroni test confirmed that PSS-exposed rats treated with saline elicited a significant decrease in time spent in open arms as compared to sham-exposed rats treated with saline (p<0.002). PSS-exposed rats treated with MDMA spent significantly more time in the open arms of the EPM as compared to PSS-exposed rats treated with saline (p<0.005). In term of time spent in closed arms, two-way ANOVA revealed a significant effect of treatment (F(1,36)=14.9, p<0.0005), and a significant PSS exposure-treatment interaction effect (F(1,36)=17.7, p<0.0002) (Figure S1B). No effects were observed for PSS-exposure or PSS-treatment interaction. Bonferroni test confirmed that PSS-exposed rats treated with saline elicited a significant increase in time spent in closed arms as compared to sham-exposed rats treated with saline (p<0.0015). PSS-exposed rats treated with MDMA spent significantly less time in the closed arms of the EPM as compared to PSS-exposed rats treated with saline (p<0.0001). In term of time spent in central platform, two-way ANOVA revealed a significant effect of treatment (F(1,36)=8.6, p<0.006) (Figure S1C). PSS-exposed rats treated with MDMA spent significantly more time in the central platform of the maze as compared to PSS-exposed rats treated with saline (p<0.05). In term of number of entries to the open arms, two-way ANOVA revealed a significant effect of PSS (F(1,36)=8.0, p<0.008), a significant effect of treatment (F(1,36)=5.6, p<0.025) (Figure S1D). No effect was observed for treatment. Bonferroni test confirmed that PSS-exposed rats treated with saline elicited a significant decrease in number of entries to the open arms as compared to sham-exposed rats treated with saline (p<0.04). No significant differences in the open arms entries between the PSS-exposed rats treated with saline or MDMA, while a trend towards a higher number of entries in the PSS-exposure treated with MDMA group was noted (p=0.08). In term of number of entries to the closed arms, two-way ANOVA revealed a significant effect of PSS (F(1,36)=14.5, p<0.0006) (Figure S1E). No effects were observed for PSS-exposure or PSS-treatment interaction. Bonferroni test confirmed that PSS-exposed rats treated with saline elicited a significant increase in number of entries to the closed arms as compared to sham-exposed rats treated with saline (p<0.0005). No differences were observed in total exploration (activity) on the maze among groups (Figure S1F).

| A | B |
| --- | --- |
|  |  |
| C | D |
|  |  |
| E | F |
|  |  |
| **Figure S1: Effect of MDMA following cue-exposure on behavioral stress responses:** All the rats were tested in the elevated plus maze (EPM) and acoustic startle response (ASR) paradigm on day 14 in unexposed controls treated with saline (Sham-PSS + Saline, n = 11) or MDMA (Sham-PSS + MDMA, n = 10), PSS-exposed animals treated with saline (PSS + Saline, n = 10), or PSS-exposed treated with MDMA (PSS-MDMA, n = 10). (**a**) Time spent in the open arms of the EPM (**b**) Time spent in the closed arms of the EPM (**c**) Time spent in the central platform of the EPM (**d**) Number of entries to the open arms of the EPM (**e**) Number of entries to the closed arms of the EPM (**f**) Overall activity in the EPM, as reflected in the total number of entries to the open and closed arms. Bars represent group means ± S.E.M. | |

**2.2. Experiment 1 - Morphology at Day 16:**

***
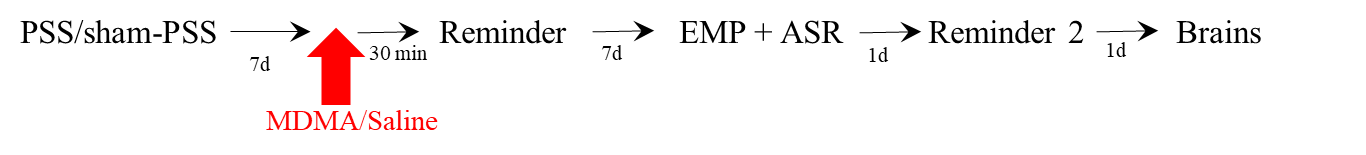
***The behavioral procedure used for the unexposed and PSS-exposed rats. Vertical arrows represent intraperitoneal injection (MDMA (5 mg/kg) or saline).

***DG granular neurons****:* Sixteen days after PSS exposure, exposed animals treated with MDMA exhibited significantly greater total dendritic length (Figure S2A) and total dendritic number (Figure S2B) as compared to exposed animals treated with vehicle (Bonferroni test: p<0.05 for both parameters) (Two-way ANOVA, a significant effect for exposure-treatment interaction effects (dendritic number F(1,32)=7.8, p<0.009 and dendritic length: F(1,32)=7.96, p<0.085). No effects were observed for PSS or treatment.

***Pyramidal neurons of the BLA:*** Exposed animals treated with MDMA exhibited significantly fewer total dendritic length (Figure S2C) as compared to exposed animals treated with vehicle (Bonferroni test: p<0.015) (Two-way ANOVA, a significant effect for treatment (F(1,33) = 13.95, p<0.00075). No effects were observed for PSS or PSS-treatment interaction. All groups showed similar dendritic number (Figure S2D).

| A | B |
| --- | --- |
|  |  |
| C | D |
|  |  |
| **Figure S2: Effect of MDMA treatment on dendritic morphology in the dentate gyrus granule neurons and pyramidal neurons of the basolateral amygdala:** (**a**) Quantitative analysis of total dendritic length (μm), and (b) total dendritic number of dentate gyrus granule cells from the suprapyramidal blade in unexposed controls treated with saline (Sham-PSS-Saline, n = 10) or MDMA (5 mg/kg) (Sham-PSS-MDMA, n = 6), PSS-exposed animals treated with saline (PSS-Saline, n = 10), or PSS-exposed treated with MDMA (PSS-MDMA, n = 10) and (**c**) Quantitative analysis of total dendritic length (μm), and **(d**) total dendritic number of pyramidal neurons of the basolateral amygdala in unexposed controls treated with saline (Sham-PSS-Saline, n = 9) or MDMA (5 mg/kg) (Sham-PSS-MDMA, n = 8), PSS-exposed animals treated with saline (PSS-SALINE, n = 10), or PSS-exposed treated with MDMA (PSS-MDMA, n = 10). | |
