# Supplementary material for: MDMA treatment paired with a trauma-cue promotes adaptive stress responses in a translational model of PTSD in rats
Source: Transl Psychiatry. 2022 May 3;12:181. doi: 10.1038/s41398-022-01952-8 (PMC9064970; doi:10.1038/s41398-022-01952-8)
Supplement: Supplementary file 3 — Supplementary Materials 3 [file 41398_2022_1952_MOESM3_ESM.docx]

**Supplementary Materials #3**

**RESULTS:**

**3.1. Experiment 2: Timely paired MDMA treatment with memory reactivation is necessary for effectiveness treatment:**

**
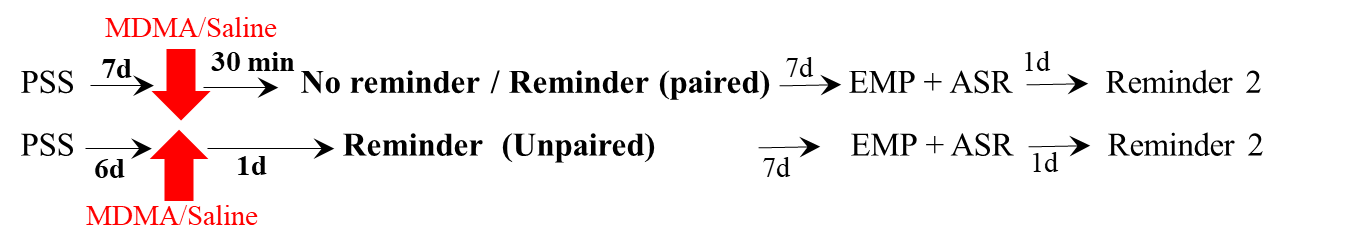
**

The behavioral procedure used for the PSS-exposed rats. Vertical arrows represent intraperitoneal injection (MDMA (5 mg/kg) or saline).

***Elevated plus maze:*** In terms of time spent in open arms, two-way ANOVA revealed a significant Treatment (F(1,48=13.4, p<0.00065|), Reminder (F(2,48)=16.7, p<0.0001) and Treatment-Reminder interaction effects (F(2,48)=9.9, p<0.0003) (Figure S2-A). In terms of time spent in closed arms, there was a significant effect of Treatment (F(1,48)=14.8, p<0.0004|), Reminder (F(2,48)=17.2, p<0.0001) and Treatment-Reminder interaction effects (F(2,48)=4.3, p<0.02) (Figure S2-B). In terms of time spent in the central platform, there was a significant effect of Treatment (F(1,48)=6.2, p<0.02), and Reminder (F(2,48)=11.0, p<0.00015) (Figure S2-B). No effects were observed for Treatment-Reminder interaction.

In terms of open arms entries, there was a significant effect of Treatment (F(1,48)=7.6, p<0.0085), Reminder (F(2,48)=21.3, p<0.0001) and Treatment-Reminder interaction effects (F(2,48)=8.8, p<0.0001) (Figure S2-D). In terms of total activity on the EPM, there was a significant effect of Reminder (F(2,48)=12.0, p<0.0001) (Figure S2-F). No effects were observed for Treatment or Treatment-Reminder interaction. Bonferroni test confirmed that PSS-exposed group treated with MDMA which paired with a trauma-cue elicited a significant increase in overall time spent in open arms and open arms entries as compared to PSS-exposed group treated with saline (p<0.0001 and p<0.0002, respectively) and to MDMA treatment unpaired with a trauma-cue (p<0.0001 and p<0.009, respectively).

| A | B |
| --- | --- |
|  |  |
| C | D |
|  |  |
| E | F |
|  |  |
| **Figure S2: The effect of MDMA treatment paired/unpaired with memory reactivation or alone on behavioral responses:** Rats were exposed for 10 min to predator-scent stress (PSS) on day 0. On day 7, rats received MDMA or Saline and 30 min later exposed to reminder for 10 min (PSS + Saline + Reminder: n=9; PSS + MDMA + reminder: n=8) or not (without reminder) (PSS + Saline alone: n=9; PSS + MDMA alone: n=10). In addition, two groups of rats were exposed to PSS and six days thereafter, MDMA or saline were administered, one day before exposure to the situational-reminder (i.e., MDMA treatment was unpaired from the reminder) (PSS + saline + Reminder: n=8; PSS + MDMA + Reminder (unpaired): n=10). (**A**) Time spent in the open arms of the EPM (**B**) Time spent in the closed arms of the EPM. (**C**) Time spent in the central platform of the EPM (**D**) Number of entries to the open arms of the EPM (**E**) Number of entries to the closed arms of the EPM (**f**) Overall activity in the EPM, as reflected in the total number of entries to the open and closed arms.  Bars represent group means ± S.E.M. | |
