# Supplementary material for: MDMA treatment paired with a trauma-cue promotes adaptive stress responses in a translational model of PTSD in rats
Source: Transl Psychiatry. 2022 May 3;12:181. doi: 10.1038/s41398-022-01952-8 (PMC9064970; doi:10.1038/s41398-022-01952-8)
Supplement: Supplementary file 4 — Supplementary Materials 4 [file 41398_2022_1952_MOESM4_ESM.docx]

**Supplementary Materials #4**

**RESULTS:**

**4.1. Experiment 3: In Lewis rats, MDMA treatment has no long-term behavioral effects**


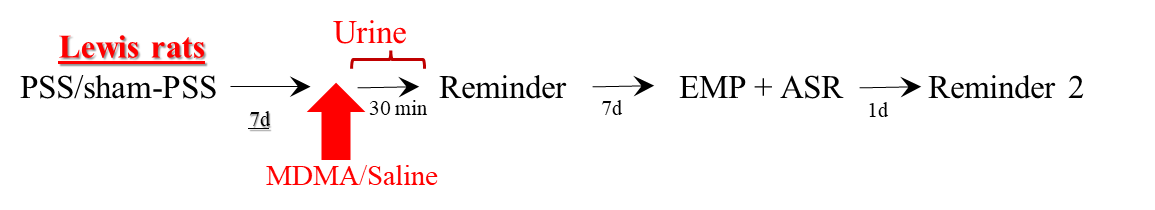


The experimental protocol: Vertical arrows represent intraperitoneal injection MDMA (5 mg/kg) or saline.

***Elevated plus maze:*** In term of time spent in open arms, two-way ANOVA revealed a significant effect of PSS-exposure (F(1,22)=36.3, p<0.0001) (Figure S4.1A). No effects were observed for treatment or exposure-treatment interaction. Post-hoc Bonferroni test confirmed that exposed groups treated with saline or MDMA elicited a significant decrease in overall time spent in open arms (p<0.003 and p<0.0015, respectively). In term of time spent in closed arms, two-way ANOVA revealed a significant effect of PSS exposure (F(1,22)=6.75, p<0.02), and a significant PSS exposure-treatment interaction effect (F(1,22)=7.36, p<0.015) (Fig.S4.1B). No effect was observed for treatment. Post-hoc Bonferroni test confirmed that exposed groups treated with saline elicited a significant increase in overall time spent in closed arms (p<0.005) (Fig.S4.1B) as compared to unexposed control treated with saline. In term of time spent in the central platform, two-way ANOVA revealed a significant effect of PSS exposure-treatment interaction effect (F(1,22)=11.7, p<0.003) (Fig.S4.1C). No effects were observed for PSS-exposure or treatment. No differences were observed in closed and open arms entries between groups (Figure S4.1D+E). All rats showed similar overall activity in the EPM (Fig.S4.1F).

| A | B |
| --- | --- |
|  |  |
| C | D |
|  |  |
| E | F |
|  |  |
| **Figure S4: In Lewis rats, MDMA treatment has no long-term behavioral effects:** The top panel **(1)** depicts the experimental protocol. Vertical arrows represent intraperitoneal injection MDMA (5 mg/kg) or Saline. Lewis rats were exposed for 10 min to predator-scent stress (PSS) or to sham-PSS on day 0. On day 7, rats received MDMA (sham-PSS + MDMA: n=6; PSS-exposed + MDMA: n=6) or Saline (sham-PSS + Saline: n=8 and PSS-exposed + Saline: n=6) and 30 min later exposed to cue-reminder for 10 min. (**A**) Time spent in the open arms of the EPM. (**B**) Time spent in the closed arms of the EPM. (**C**) Time spent in the central platform of the EPM. (**D**) Number of entries to the open arms of the EPM. (**E**) Number of entries to the closed arms of the EPM. (**F**) Overall activity in the EPM, as reflected in the total number of entries to the open and closed arms.  Bars represent group means ± S.E.M. | |
