# Supplementary material for: MDMA treatment paired with a trauma-cue promotes adaptive stress responses in a translational model of PTSD in rats
Source: Transl Psychiatry. 2022 May 3;12:181. doi: 10.1038/s41398-022-01952-8 (PMC9064970; doi:10.1038/s41398-022-01952-8)
Supplement: Supplementary file 5 — Supplementary Materials 5 [file 41398_2022_1952_MOESM5_ESM.docx]

**Supplementary Materials 5**

**RESULTS:**

# **Experiment 4: The glucocorticoid receptor and the serotonin receptors 5-HT-_1A_ and 5-HT-_2A_ are necessary for the anxiolytic effects of MDMA treatment:**

***
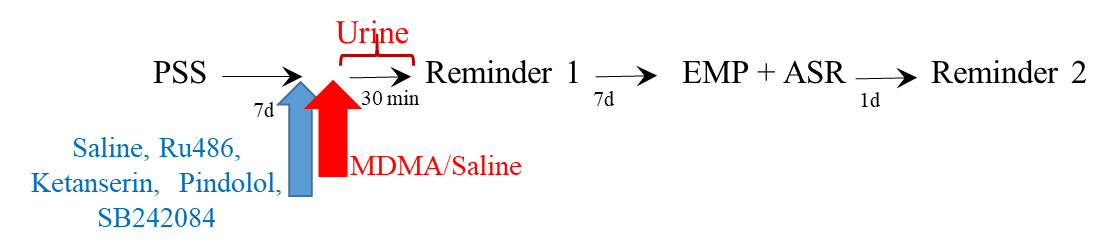
***

***Elevated plus maze:*** Rats exposed to PSS and pre-treated with RU486, Ketanserin or Pindolol in addition to MDMA spent significantly less time in the open arms of the EPM (p<0.009, p<0.04, p<0.0015, respectively; F(4,25)=7.6, p<0.0004, (Figure. S5A) and in the central platform of the maze (p<0.0001 for all groups; F(4,25)=20.0, p<0.0001, (Fig. S5C), and entered the open arms less frequently (p<0.0001 for all groups; F(4,25)=23.4, p<0.0001, (Fig. AAc) than the saline-treated MDMA injection group (p<0.0008, p<0.002, p<0.00035, respectively; F(4,25)=9.9, p<0.0001, (Fig. AAc). In addition, rats exposed to PSS and pre-treated with RU486, ketanserin or Pindolol in addition to MDMA spent significantly more time in the closed arms (p<0.0001 for all groups, F(4,25)=15.0, p<0.0001, (Fig. XXB). All rats showed similar overall activity in the EPM (Fig. XXc). On the other hand, pretreatment with or SB242084 did not prevent MDMA-induced these behavioral changes.

| A | B |
| --- | --- |
|  |  |
| C | D |
|  |  |
| E | F |
|  |  |
| **Figure S5:** **The glucocorticoid receptor and the serotonin receptors 5-HT-_1A_ and 5-HT-_2A_ are necessary for the anxiolytic effects of MDMA treatment:** All the rats were tested in the elevated plus maze (EPM) and acoustic startle response (ASR) paradigm on day 14 in unexposed controls treated with saline (Sham-PSS + Saline, n = 11) or MDMA (Sham-PSS + MDMA, n = 10), PSS-exposed animals treated with saline (PSS + Saline, n = 10), or PSS-exposed treated with MDMA (PSS-MDMA, n = 10). (**a**) Time spent in the open arms of the EPM (**b**) Time spent in the closed arms of the EPM. (**c**) Time spent in the central platform of the EPM (**d**) Number of entries to the open arms of the EPM (**e**) Number of entries to the closed arms of the EPM (**f**) Overall activity in the EPM, as reflected in the total number of entries to the open and closed arms. Bars represent group means ± S.E.M. | |
